# Functional interrogation of autoimmune disease genetics using CRISPR/Cas9 technologies and massively parallel reporter assays

**DOI:** 10.1007/s00281-021-00887-4

**Published:** 2021-09-10

**Authors:** James Ding, Antonios Frantzeskos, Gisela Orozco

**Affiliations:** 1 Centre for Genetics and Genomics Versus Arthritis, Division of Musculoskeletal and Dermatological Sciences, School of Biological Sciences, Faculty of Biology, Medicine and Health, The University of Manchester, AV Hill Building, Oxford Road, Manchester, M13 9LJ UK; 2 NIHR Manchester Biomedical Research Centre, Manchester University NHS Foundation Trust, Manchester Academic Health Science Centre, Manchester, M13 9WL UK

**Keywords:** Autoimmune disease, CRISPR/Cas9, MPRA, Functional genetics

## Abstract

Genetic studies, including genome-wide association studies, have identified many common variants that are associated with autoimmune diseases. Strikingly, in addition to being frequently observed in healthy individuals, a number of these variants are shared across diseases with diverse clinical presentations. This highlights the potential for improved autoimmune disease understanding which could be achieved by characterising the mechanism by which variants lead to increased risk of disease. Of particular interest is the potential for identifying novel drug targets or of repositioning drugs currently used in other diseases. The majority of autoimmune disease variants do not alter coding regions and it is often difficult to generate a plausible hypothetical mechanism by which variants affect disease-relevant genes and pathways. Given the interest in this area, considerable effort has been invested in developing and applying appropriate methodologies. Two of the most important technologies in this space include both low- and high-throughput genomic perturbation using the CRISPR/Cas9 system and massively parallel reporter assays. In this review, we introduce the field of autoimmune disease functional genomics and use numerous examples to demonstrate the recent and potential future impact of these technologies.

## Introduction—the case for functional interrogation

Autoimmune diseases are diverse and numerous, including rare monogenic disorders such as Aicardi-Goutières syndrome and highly prevalent disorders with a complex genetic contribution to risk, such as rheumatoid arthritis (RA). In the US population, the prevalence of autoimmune diseases is estimated at over 3% of the population and, whilst some individual diseases do not follow these rules, this prevalence is higher amongst women and increases with age [1]. Amongst women, autoimmune diseases are one of the leading causes of death in populations of European ancestry [2, 3].

From a pathophysiological perspective, these diseases share a common mechanism, being caused by inappropriate or dysregulated immune responses. However, depending on the disease-specific context, this can lead to an array of different clinical manifestations. When focussed on patient care, it may seem that there is little to be gained from considering these diseases as one group; however, the involvement of common biological pathways presents shared therapeutic targets. For example, whilst first used in the treatment of RA, anti-tumour necrosis factor therapies are used in many autoimmune diseases characterised by chronic inflammation, including Crohn’s disease (CD) and inflammatory bowel disease (IBD) [4].

Whilst many therapeutic options have already been identified, these typically are effective only for a proportion of patients, such that there is a need for novel therapies. Genetic studies, identifying mutations or variants associated with disease, can highlight genes or pathways as potential therapeutic targets. Drugs supported by genetic evidence are estimated to be twice as likely to proceed through clinical development as those without [5].

The high prevalence of complex genetic autoimmune diseases has enabled population-based genome-wide association (GWA) studies. A series of GWA studies published by the Wellcome Trust Case Control Consortium comprise the foundation of the genetic data available for many autoimmune diseases and have identified hundreds of associated loci [6–15]. These studies have demonstrated that there is a common genetic background predisposing to autoimmunity, with disease-specific genetic associations and environmental factors determining the resulting clinical manifestations [16].

A small number of loci, such as those overlapping the major histocompatibility complex (MHC), carry a disproportionate proportion of heritability and have relatively large effect sizes. Here, the mechanism by which individuals are predisposed to disease can be quite clear. For example, a nonsynonymous, deactivating single nucleotide polymorphism (SNP) in the coding sequence of *tyrosine kinase 2* (*TYK2*) is associated with protection from many autoimmune diseases. As a consequence of this discovery, a TYK2 inhibitor is currently being trialled for use in treating patients with CD, systemic lupus erythematosus (SLE) and psoriasis (Ps), with promising initial results [17].

The majority of loci identified through GWA studies as being associated with autoimmune diseases are found in noncoding regions [18]. What is more, as a result of linkage disequilibrium, the resolution with which an association can be mapped is often limited to a large number of co-inherited SNPs. For these loci, it is often unclear how disease susceptibility is conferred, such that the promise of genetic data leading to therapeutic discoveries has yet to be fully realised. Here, functional genomics has the potential to bridge the gap between genetic data and therapeutic translation, from bench to the bedside.

For a given locus, genetic fine-mapping and functional annotation are often helpful in prioritising a small number of highly credible SNPs that are more likely to be causal (high posterior probability). For many loci, however, it is impossible to resolve associations to a single highly credible SNP or functional element. Furthermore many loci associated with autoimmune disease do not overlap with expression quantitative trait loci (eQTLs) [19], which could implicate a causal gene. For many loci, it is unclear which protein-coding genes are affected by disease-associated variants, let alone in what way, in what context and through what mechanism.

Large collections of epigenomic data from disease-relevant cell types have enabled bioinformatic approaches to link disease-associated variants to putative causal genes. For example, this has been achieved by correlating the accessibility of underlying genetic elements with the transcription of proximal genes [20] or by incorporating chromatin conformation capture data into a model of enhancer activity [21]. Such predictions and the underlying data generate valuable hypotheses for individual loci, which require experimental validation.

In this review, we will discuss methods used to interrogate loci associated with autoimmune disease susceptibility, establishing causal variants, implicated regulatory elements and relevant protein-coding genes. In particular, we will focus on application of CRISPR/Cas9 technologies and massively paralleled reporter assays (MPRAs), highlighting publications where these techniques have been used to great effect in the study of autoimmune diseases or exemplar publications in other diseases. These examples are summarised in Table 1.

**Table 1:** A summary of autoimmune disease risk loci used as examples in this review

Locus	Disease(s)	Gene	Approach	Findings	Ref
1p13	RA, SLE, T1D et al	*PTPN22*	Transgenic knock-out model	Deficiencies in T cell function and development	[22]
6q23	CeD, IBD, RA, SLE, T1D et al	*TNFAIP3*	Targeted deletion in T-helper cells, following discovery of allele-specific effects in T-helper cell MPRA targeting 14 loci associated with a total of 10 autoimmune diseases	Reduced *TNFAIP3* expression and hyperactivation upon stimulation	[28]*
10p15	RA, T1D et al	*IL2RA*	Transgenic models, targeted deletion and SNP editing, following identification of regulatory element in T-helper like cell line CRISPRa screen	Delay in IL-2RA expression upon activation of naïve T-cells	[27]
5p13	Ankylosing spondylitis	*PTGER4*	SNP editing in lymphoblastoid cell line, following lymphoblastoid cell line MPRA targeting SNPs in LD with known eQTLs	Decrease in *PTGER4* expression	[37]
5q33	SLE	*miR-146a*	CRISPRi, CRISPRa and targeted deletion in monocyte-like cell line, as well as targeted deletion in monocytes	Decreased NFκB binding and downregulation of miR-146a	[44]†

^*^Transgenic knock-out models of TNFAIP3 also discussed [24, 25]. †SNP of interest identified in an MPRA targeted 91 SLE loci[41]

## Genome editing

Genome editing has long been used as a means of studying the consequences of disease-associated variants. For example, where a protein-coding gene is strongly implicated, knock-out models have been used to determine the consequences of deficiency in this gene and their potential relevance to disease. Such is the case for *protein tyrosine phosphatase, non-receptor type 22* (*PTPN22*), which is strongly associated with many autoimmune diseases. Mice deficient in PTPN22 have abnormalities in T cell function and development [22]. Similarly, mouse knock-out models have been used to establish a role for *TNF alpha-induced protein 3* (*TNFAIP3*) in systemic autoimmunity [23]. Complete loss of *TNFAIP3* in mice led to multi-organ inflammation [24, 25], whilst tissue-specific knockout in dendritic cells, for example, amplified B and T cell activation [25], whilst knockout in macrophages resulted in inflammatory cytokine secretion [24].

**Fig. 1:**
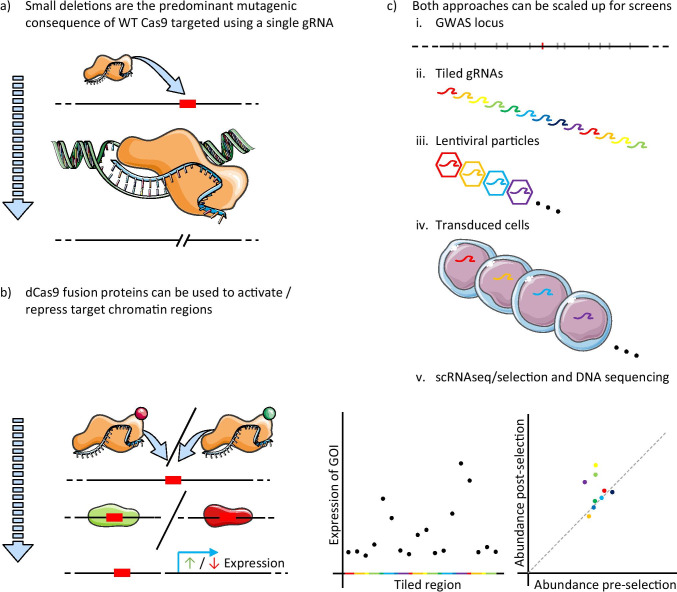
CRISPR/Cas9-based methods. When targeted to a region of interest (red box) by a single gRNA (blue ribbon), Cas9 (orange) predominately generates small deletions of a few base pairs (**a**); in coding regions, this is likely to knock out a gene. By fusing chromatin modifiers (red and green circles) to a catalytically inactivated Cas9 protein (dCas9), it is possible to activate or repress regions of interest (**b**), potentially switching on or off regulatory elements and downstream genes of interest (GOI). In much the same way as for MPRAs, it is possible to tile gRNAs and use lentiviral methods to scale up both of these approaches (**c**). Screens can be analysed using single-cell RNA sequencing (scRNA-seq), or more classically by measuring the abundance of gRNAs following selection (e.g. drug resistance or cell sorting based on a GOI)

As genome editing methods have improved, this experimental approach has become feasible even for non-coding regions. The most significant development in this area has been the discovery of the CRISPR/Cas9 system and its development for genome engineering (reviewed in [26]). In brief, in its canonical form, the CRISPR/Cas9 system consists of a guide RNA (gRNA) targeted endonuclease (Cas9) capable of generating double-strand breaks at desired genomic loci with high efficiency and specificity. These breaks are repaired by the cell’s DNA repair pathways and successive rounds of efficient cleavage and repair are only escaped when targeted Cas9 is no longer present or an error occurs during repair, such that the target site is disrupted (Fig. 1).

Experiments performed by Simeonov et al. focussed on rs61839660 represent a relevant example of canonical CRISPR/Cas9-mediated genome editing [27]. This non-coding variant is found within an intron of *interleukin-2 receptor alpha* (*IL2RA*) and is associated with multiple autoimmune diseases, including type 1 diabetes (T1D) and RA. The local sequence is highly conserved between human and mouse, such that B6 mice are homozygous for the protective allele. Deletion of 12-bp surrounding rs61839660, achieved through CRISPR/Cas9 cleavage and non-homologous end joining (NHEJ), caused a reduction in the number of naïve T-cells that express IL-2RA following 1 day of ex vivo activation. The researchers went on to show that replacing the protective allele with the risk allele, achieved through CRISPR/Cas9 cleavage and homology directed repair (HDR)-mediated repair using an exogenous template, caused the same phenotype that is due to a delay in IL-2RA activation.

Whilst the above example is focussed on transgenic mice, such approaches are also possible in primary human cells. For example, Bourges et al. used multiple guide RNAs to generate deletions ranging from 18 to 50-bp surrounding rs6927172 in primary human T-helper cells [28]. This variant is intergenic, located approximately 200 kb from *TNFAIP3*, and is associated with RA, coeliac disease (CeD), IBD and 8 other autoimmune diseases. Upon activation, cells harbouring these deletions had reduced *TNFAIP3* expression and showed increased expression of activation marker *cluster of differentiation 69* (*CD69*), as well as increased secretion of cytokines interleukin (IL) 4, IL-17 and interferon gamma (IFN-ɣ).

Whilst much more feasible than with previous technologies, canonical CRISPR/Cas9-mediated genome editing remains labour intensive and low throughput. It is, therefore, generally used only to validate other experimental data or following preliminary experimentation. Such was the case in both of these instances, where preliminary experiments had enabled researchers to focus on a single highly credible SNP. In order to achieve this, higher-throughput methodologies are required and the methods implemented by Simeonov et al., Bourges et al. and others are discussed later in this review.

One approach to increasing the throughput of these experiments is merely to perform them in an arrayed context. Whilst lower throughput than pooled CRISPR screens, this offers more opportunity to assess multiple phenotypes that may be affected by individual gRNAs. For example, in order to map a network of regulatory genes and targets, Freimer et al. used an arrayed approach to knockout (KO) 50 genes found to regulate *cytotoxic T-lymphocyte-associated protein 4* (*CTLA4*), *IL2RA* and *IL-2*. The effect of these knockouts was assessed by RNA sequencing (RNA-seq) and assay for transposase accessible elements sequencing (ATAC-seq) [29]. It is hoped that the regulatory network established by Freimer et al. may identify further potential drug targets in addition to *CTLA4*, *IL2RA* and *IL-2*.

## Massively parallel reporter assays

**Fig. 2:**
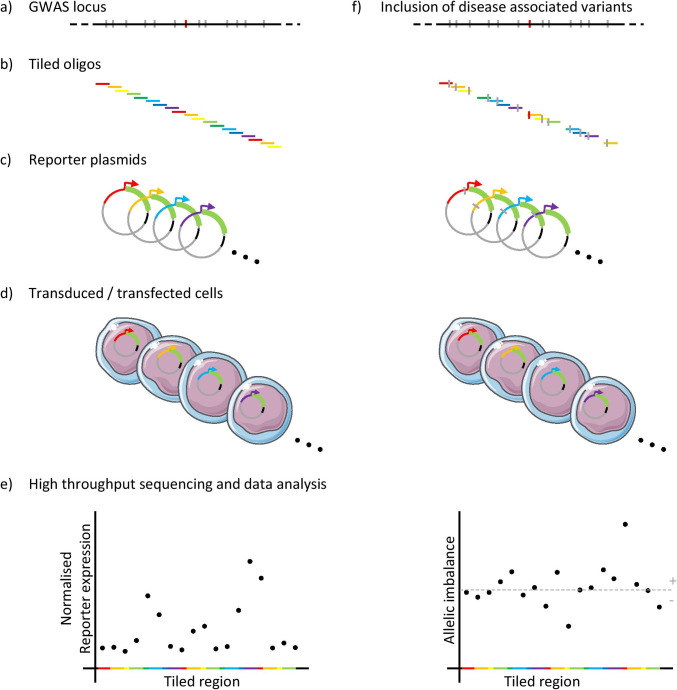
Experimental overview of massively parallel reporter assays (MPRAs). MPRAs can be targeted to GWAS loci (**a**) comprised of an index variant (red) and all credible co-inherited variants (grey). Oligos are synthesised across the entire region of interest (**b**) and cloned into reporter plasmids containing a minimal promoter, reporter gene and unique barcode (**c**). Plasmids are delivered to a disease-relevant cell type (**d**) and the effect of the synthesised oligos on reporter gene transcription determined. Generally reporter transcripts, quantified by RNA sequencing, are assigned to individual barcodes/oligos and the prevalence of these barcodes/oligos in a DNA-based library is used for normalisation (**e**). In addition to identifying potential enhancer elements (**a**–**e**), MPRAs can be designed to include oligos with both risk and protective alleles for variants of interest, thereby characterising allele-specific enhancer activity (**f**)

MPRAs can be employed to evaluate the regulatory potential of disease-associated loci in a high-throughput manner. These assays use a library of synthesised candidate sequences which are cloned into a reporter vector containing a promoter, reporter and unique barcode. High-throughput sequencing and deconvolution of barcodes are used to measure the influence of candidate regions on transcription, with the prevalence of the barcode in RNA being compared to that in DNA [30–32] (Fig. 2). MPRAs can be carried out either in vivo or in vitro (both primary and immortalised cell lines) and are most commonly delivered either by plasmid, or a viral vector (adeno-associated virus (extragenomic) or lentivirus (intragenomic)) [33–35].

MPRAs are commonly applied to explore various regulatory elements, including transcription binding motifs, post-transcriptional regulatory elements and eQTLs. They can be designed to dissect a specific motif or an entire region at a base pair level through multiple perturbations (saturation mutagenesis) to identify the role (activating/repressing/none) of each base pair within that region [34, 36, 37]. More commonly when dealing with GWAS variants of unknown function, MPRAs will be designed to compare thousands of regions containing either protective or risk GWAS SNPs [31, 38]. An example of the scale MPRAs can reach was demonstrated by van Arensbergen et al. who developed a plasmid-based MPRA technology known as survey of regulatory elements (SuRE) which aided by high-throughput sequencing can screen millions of SNPs and examine regions up to 2 kb in length [39].

MPRAs have proved to be a valuable tool for prioritising risk variants that originate from GWA studies. One such study examined 2756 variants in LD with 75 lead SNPs associated to red blood cell traits. A total of 32 variants were found to affect expression in K562 cells, providing a reduced list of candidate variants for functional interrogation in this cell line. Several SNPs were followed up with CRISPR/Cas9-induced deletions. For example, deletion of rs737092 resulted in the modulation of *RNA binding motif protein 38* (*RBM38*) and *ribonucleic acid export 1*. *RBM38* KO demonstrated that this gene played a role in the regulation of alternative splicing in human erythropoiesis [40]. In a similar approach, after establishing the specificity of an MPRA-based strategy by targeting variants in strong LD with eQTLs, Tewhey et al. examined over 9000 variants in 163 GWA loci. The MPRA identified 248 variants with allelic expression differences. An identified functional SNP rs9283753 in LD with a lead SNP for ankylosing spondylitis was edited using a CRISPR/Cas9 HDR approach to produce a homozygous cell line for that variant and confirmed its regulatory role by demonstrating it led to a decrease in the *prostaglandin E2* (*PTGER4*) gene [37].

There are few MPRA studies focusing specifically on autoimmune disease-associated loci, recently Lu et al. screened all 3073 GWAS-linked systemic lupus erythematosus (SLE) variants at 91 loci in lymphoblastoid and T-cell like cell lines: GM12878 and Jurkat. Fifty-one variants showed allele-specific activity in the B cell line and the authors also characterised altered binding of transcription factors (TFs) in particular *nuclear factor kappa-light-chain-enhancer of activated B cells* (*NFκB*)-related TFs. Moreover 92 allele-specific enhancer variants were identified in the T cell line, 25% of which were shared with the B cell line [41]. A follow-up study focussed on SNP rs2431697, identified in the aforementioned MPRA, used CRISPR-induced HDR to produce clones homozygous for the reference allele and the risk variant. The risk variant showed a decrease of NFκB binding and resulted in miR-146a being downregulated, something that has been observed in SLE patients [42].

One study recently applied MPRA in primary T-helper cells for the first time in order to focus on immune-disease-related loci, given the enrichment of autoimmune disease-linked SNPs in T-helper cell regulatory regions [28]. This strategy was successful in validating causality to a single SNP in loci that were well fine-mapped. For example, an IBD locus fine mapped to a single SNP rs1736137 [43] led to allelic-specific expression differences in both stimulated and unstimulated cells. The same study demonstrated that out of three SNPs with a sum total posterior probability of 99% in AS (ankylosing spondylitis) only one of the three was found to affect expression. Furthermore, in regions with a greater number of candidate SNPs, the MPRA was capable of prioritising a single SNP in an IBD and MS-linked locus out of 44 candidate SNPs and of discovering a synergistic effect between two SNPs in a T1D locus that showed a greater effect when together as opposed to individually. Bourges et al. examined the *TNFAIP3* locus which has been linked with multiple autoimmune diseases including RA, SLE and IBD. In this study, they focused on the fine-mapped IBD-related SNPs [43] and showed rs6927172 was the primary candidate resulting in reduced expression of *TNFAIP3* and also reduced NFκB binding, which has previously been associated with super-enhancer formation [44].

When studying MPRA results, it is important to consider some of the limitations this type of reporter assay has. MPRAs generally utilise plasmid DNA which does not consider the native architecture of chromatin; chromatin interactions will not be recapitulated, nor will nearby TF motifs [45]. Lentiviral MPRA methods have been developed in order to introduce the plasmid into the genome, which saw more reproducible results than the plasmid-based method [46]. However, lentiviral-based MPRAs still do not reproduce the endogenous genomic context of the studied SNPs [33]. Moreover, most of the aforementioned studies only study a limited number of cell lines, again potentially missing the disease-relevant context in which a SNP plays a functional role. MPRAs alone cannot determine the causality of a SNP and cannot link the SNP to a gene; they are, therefore, best used as a primary screen to reduce the number of candidate SNPs.

Kreimer et al. meta-analysis of MPRAs focused on regions overlapping various observed regulatory elements and identified chromatin accessibility as the best predictive marker for an enhancer and histone 3 lysine 27 acetylation (H3K27ac) as the best predictive chromatin mark [47]. This matches well with our understanding of enhancers based on other techniques and demonstrates the relevance of data originating from MPRAs. However, an unbiased approach by Kheradpour et al. (i.e. observed regulatory marks were not preselected for) showed that even regions where no regulatory mark was apparent could lead to a difference to promoter activity [31]. It is therefore important to consider that MPRAs may generate false positives (low specificity), as the influence of candidate regions is assessed in a highly artificial context. An alternative interpretation could moot that MPRAs offer a very high level of sensitivity; Benton et al. observed eleven different enhancer predicting annotations (including DNase I hypersensitive sites, H3K27ac, H3K4Me1 and p300 binding sites) in four different cell types and found that a significant amount of functional enhancers are overlooked when focusing exclusively on enhancer predictive annotations [48].

## CRISPR screens

Pooled CRISPR screens are typically carried out by introducing a large number of gRNAs into cells using viral methods, with a low multiplicity of infection, such that each cell receives only a single guide. The identity of this guide is determined by sequencing following selection for a phenotype of interest, or when generating transcriptomic information from single cells [49]. Wild-type Cas9 is either delivered transiently in addition to the gRNA, or an engineered cell line capable of constitutive/inducible Cas9 expression is used. In this instance, gRNA targeted Cas9 will predominantly generate small deletions of several nucleotides around their targeted cleavage site. Alternatively, a catalytically inactivated Cas9 molecule (dCas9) fused to various effector molecules can be used, for example VP64 to activate (CRISPRa), or KRAB to repress (CRISPRi) chromatin at the target site [50]. These technologies enable large-scale genetic screening of regulatory elements and GWAS-associated non-coding variants in both cell lines and primary cells [51].

CRISPR screens, similarly to MPRAs, have been utilised to provide information regarding non-coding functional elements. This is achieved either by targeting specific elements of interest or by tiling entire regions of interest with gRNAs in an unbiased approach [52–56]. Canver et al. were one of the first groups to attempt tiling gRNAs over non-coding DNAse hypersensitivity sites (DHSs) in a pooled screen aiming to identify intronic *B-cell lymphoma/leukaemia 11A* (*BCLL11A*) enhancers in the HUDEP-2 (human erythroid progenitor) cell line. Foetal haemoglobin (HbF) was used as an output phenotype as it is known that *BCL11A* reduction leads to an increase in HbF. In contrast to MPRAs, this methodology ensured that, within reason, the genetic and epigenetic context of the underlying sequence was maintained in addition to ensuring that any readout was related to a specific gene and pathway. In one of the DHSs, they found a 42-bp region targeted by 10 different gRNAs that resulted in the most significant change in HbF [57]. Further direct comparison of MPRAs and CRISPR screens is provided in Table 2.

**Table 2:** Summary of key attributes of MPRAs and CRISPR screens. Additional details, such as throughput, cost and time, are omitted as they are widely variable and largely overlapping, depending largely on the precise experimental design

	MPRAs	CRISPR screens
Methodology	Fragments of interest inserted in reporter plasmid, with the effect of individual fragments on expression inferred from their abundance in reporter transcripts	WT or modified Cas9 targeted to genomic loci of interest using gRNAs, with the impact of individual gRNAs on a specific phenotype inferred by their abundance following selection for that phenotype.^a^
Strengths	• Potential to directly infer allele-specific effects	• Retention of genomic context • Any selectable phenotype can be used in addition to single-cell approaches
Limitations	• The size of fragments of interest that can be inserted is limited • Susceptible to false positives in enhancer discovery, as the genomic context of individual fragments is lost • Reporter plasmid design requires optimisation for different cell types	• Limited resolution given that gRNAs can be designed to a limited proportion of the genome • Susceptible to false negatives as gRNAs have variable efficacy • The delivery of components, especially modified Cas9 molecules to many cell types is limiting^b^

^a^In single-cell RNA sequencing-based CRISPR screens, the transcriptional profile of cells with individual gRNAs is measured. ^b^Delivery of reporter plasmids is also limiting, but to a lesser extent

Simeonov et al. applied CRISPRa screening to interrogate autoimmune disease susceptibility loci associated with *CD69* and *IL2RA*. Tiling CRISPR/dCas9-VP64 in Jurkats over the risk region and sorting cells based on expression of either CD69 or IL2RA led to the identification of potential regulatory elements which they referred to as CRISPRa-responsive elements (CaRE). Three such sites were identified in the *CD69* locus and 6 in the *IL2RA* locus, one of which contains the autoimmunity risk variant rs61839660. It was this CaRE that formed the focus of their genome editing work in B6 mice that has already been described [27].

Fulco et al. utilised the complementary CRISPRi method to examine the autoimmunity-linked *MYC* locus and *GATA1* region employing 98,000 gRNAs which successfully detected enhancers for those genes [52]. The same group coupled CRISPR/dCas9-KRAB with RNA fluorescence in situ hybridization (FISH) and flow cytometry (CRISPRi-FlowFISH), fluorescently labelling candidate gene transcripts in gRNA transduced cells that are then sorted based on expression levels. CRISPRi-FlowFISH was used to target all DHSs near selected genes of interest and measure their expression, revealing single regulatory elements regulating multiple genes and conversely an individual gene being regulated by several enhancers in K562 cells. The group subsequently developed an activity-by-contact (ABC) model that combined DHS and H3K27ac occupancy with chromosomal interaction data to predict enhancer-gene links [58]. The ABC model was used to develop enhancer-gene connections in 74 different cell types. ABC enhancers were enriched for GWAS variants and the model was able to link IBD credible SNPs to known IBD-linked genes like *IL10*, as well as linking new genes such as *peptidyl-prolyl cis–trans isomerase* (to SNP rs1250566). Novel links generated using their model were validated with CRISPRi-FlowFISH [21].

The aforementioned CRISPRa/i screens rely on a cell line constitutively expressing dCas9. Previous studies in primary cells focused on transgenic mice expressing Cas9 or dCas9 for screening murine immune cells or used CRISPR/Cas9 ribonucleoproteins (RNP) to drive HDR or NHEJ in primary human cells. Both murine and human primary immune cell gene KO screens have been helpful in investigating immune-related circuitries [51, 59–65]. A primary murine T cell CRISPR KO screen, performed by Henriksson et al., examined genes involved in the activation and differentiation of murine T-helper type 2 cells (Th2). In combination with ATAC-seq and ChIP-seq for key TFs, the screen showed a significant overlap in genes regulating differentiation and activation and revealed known genes like *signal transducer and activator of transcription 6* (*Stat6*) as well as identifying new Th2 regulating genes [65]. Whilst another group optimised CRISPR RNP delivery in murine innate immune cell types [66], Shifrut et al. developed single guide RNA lentiviral infection with Cas9 protein electroporation (SLICE) to employ a genome-wide CRISPR screen in human primary cytotoxic T cells utilising 77,441 gRNAs targeting 19,114 genes to identify genes with a role in proliferation following stimulation [51]. Selected hits from the screen were examined in-depth by CRISPR perturbation this time combined with single-cell RNA sequencing in both stimulated and unstimulated cells. The same technique was used in a recent study that took a different approach, initially inspecting upstream regulators of 3 genes vital for immune-related pathways, *CTLA4*, *IL2RA* and *IL-2*. This was followed by KO of 24 identified upstream regulators to investigate the downstream gene networks [29]. Similar CRISPR-based perturbation assays have also been applied in primary T-regulatory (Treg) cells targeting selected TFs in various cytokine conditions to reveal regulators of *forkhead box P3*, *CTLA-4* and *IFN-ɣ* to help understand Treg states [64]. Only recently has delivery of dCas9 fusion proteins been accomplished in primary T cells (with the dCas9 protein being delivered using a lentiviral vector) [67]. At the time of writing, the study is yet to be peer reviewed. The authors use both CRISPRa and CRISPRi to map *IL-2* and *IFN-ɣ* gene networks.

CRISPR methods come with some limitations. Cas9 binding is restricted by its PAM site, making it challenging to target all candidate sites [68], especially complex, repetitive regions such as the major histocompatibility complex. Highly repetitive regions are also less amenable to MPRAs, but to a lesser extent. Moreover, CRISPR-based techniques are associated with potential off-target effects that need to be controlled for, for example by validating results with additional gRNAs [69]. CRISPR-associated proteins from other bacterial species with different PAM sites have been developed, in addition to engineering of the widely used *Streptococcus pyogenes* Cas9, in order to increase the percentage of the genome that can be targeted, improve efficiency and reduce off-target effects. CRISPR screens unlike MPRAs cannot currently be utilised for saturation mutagenesis; however, this may be possible with newly developed technologies such as prime [70] and base editing [71]. Base editors (BEs) utilise Cas9-nickase, which only cuts a single strand, or dCas9 fused to a deaminase. Currently two types of BEs exist, cytosine and adenine base editors which can convert C → T or A → G, respectively [71]. Prime editing also makes use of Cas9-nickase but is fused to a reverse transcriptase which uses the specifically designed prime editing gRNA, which contains the desired edit, as a template to be copied into the genome. Both these new technologies improve on single base substitution efficiency by overcoming the need for HDR-dependant repair following DSBs [70]. Whilst not yet used in the context of autoimmune diseases, BEs have recently been applied in a screen for the first time targeting the *BRCA1* coding region to determine the function of variants of unknown significance [72]. Prime editing is a more recent technology and has not been developed for high-throughput assays yet; however, it is likely to be a dominant tool in future functional studies.

The strength of screens and CRISPRi in particular to prioritise disease-related non-coding variants was demonstrated by Ray et al. They compared seven assays including perturbational assays: CRISPRa-FlowFISH and CRISPRi-FlowFISH; observational assays: DNase I-seq, ATAC-seq and ChIP-seq (observing H3K27ac); and both lentiviral and transfection-based MPRAs targeting all common variants at the autoimmune-associated *TNFAIP3* locus, determining to what extent each assay enriches for disease-linked SNPs and thus how well the assay could prioritise causal SNPs [46]. Using all of these methods in T-cell, B-cell and monocyte cell lines, they found that GWAS SNPs were only enriched amongst accessible chromatin (ATAC-seq peaks) and regions of chromatin that when targeted by CRISPRi lead to a change in *TNFAIP3* expression. It is important to note that CRISPRi gRNAs were only designed to regions of accessible chromatin and that not all variants determined to be regulatory overlapped between assays. This emphasises the variability of assays in different contexts, the necessity to combine multiple assays in different cell lines to prioritise disease-linked variants and the benefit of combining both observational and perturbation methods.

## Conclusions

Understanding the genetic mechanisms underlying autoimmune diseases is still a major challenge. Appropriately characterising GWAS SNPs in different cell types and contexts can assist in defining relevant genes and pathways. Advances in screening techniques of high-throughput perturbation and genome editing have allowed examination of non-coding autoimmune-associated SNPs and immune-related pathways at scale, prioritising certain SNPs that can then be studied thoroughly, including at the single base pair level in order to establish causative variants and genes.

Integrating epigenetic data with risk SNPs has helped to identify relative cell types, contexts that variants are likely to act in and potential target genes [73, 74]. The EpiMap project, for example, correlated epigenomic marks and gene expression data from a large number of samples in order to predict tissue-specific links between enhancer and genes [20].

To date, variant interrogation has been predominantly carried out in cell lines. Recent efforts by various groups have optimised the same techniques used in cell lines in some of the more relevant primary human and murine immune cell types; however, a greater range of primary cell types is likely required in order to accurately model all autoimmune disease associations.

Functional screens have helped identify causal variants and improved our understanding of immune networks. Whilst novel autoimmune therapies are yet to arise as a direct consequence of CRISPR or MPRA-based screens, CRISPR screens in cancer have demonstrated their utility in identifying therapeutic targets. For example, a genome-wide loss-of-function screen examining the β-catenin signalling pathway identified KMT2A as a potential target and in vitro validation confirmed that two KMT2A-menin suppress β-catenin–active colorectal cancer cells [75].

Both CRISPR screens and base pair editing methods will benefit from the current focus and rapid development of CRISPR technology. Whilst they have not attracted the same level of focus, it is clear that MPRA methods also have great potential for improvement, including extending the range of disease-relevant primary cell types used, improving the reporter plasmids used and increasing the size of region that can be incorporated.

Currently a minority of GWAS autoimmune SNPs have been fully characterised. Just as there have been major advances in the availability of epigenetic data for generating testable hypotheses and in the tools available in order to test these in a low-throughput manner, MPRAs and CRISPR screens have emerged as informative and tractable approaches to interrogating autoimmune disease genetics. Further application of these methods, along with incremental improvement, and careful curation and integration of the resulting data will undoubtedly lead to an improved understanding of autoimmune diseases.

## Data Availability

Not applicable.
